# Atypical hemolytic uremic syndrome with a *C3* variant following COVID-19: a case report

**DOI:** 10.3389/fped.2025.1507727

**Published:** 2025-01-23

**Authors:** Masato Ando, Kazuo Kubota, Saori Kadowaki, Minako Kawamoto, Norio Kawamoto, Haruka Okamoto, Soichiro Nagaya, Yuki Miwa, Hidenori Ohnishi

**Affiliations:** ^1^ Department of Pediatrics, Graduate School of Medicine, Gifu University, Gifu, Japan; ^2^ Clinical Genetics Center, Gifu University Hospital, Gifu, Japan; ^3^ Gifu University Advanced Critical Care Center, Gifu, Japan

**Keywords:** atypical hemolytic uremic syndrome, GOF *C3* variant, severe acute respiratory syndrome coronavirus 2, alternative complement pathway, inborn errors of immunity

## Abstract

Atypical hemolytic uremic syndrome (aHUS) is a form of thrombotic microangiopathy (TMA) characterized by the triad of microangiopathic hemolytic anemia, thrombocytopenia, and acute kidney injury, and is caused by overactivation of the alternative complement pathway. A 13-year-old Japanese boy with an unremarkable medical history developed symptoms of TMA following coronavirus disease 2019 (COVID-19) infection with mild respiratory symptoms. He was eventually diagnosed with aHUS with a gain-of-function *C3* variant. He improved with supportive therapy and plasma exchange, and did not require anti-C5 antibody therapy. In the literature, more than 20 cases of *de novo* or relapsed aHUS have been described following COVID-19. It has been shown that the complement lectin pathway can be activated by the severe acute respiratory syndrome coronavirus 2 (SARS-CoV-2) spike and N proteins, and the alternative pathway can be activated by the SARS-CoV-2 spike protein. The current case highlights the possibility that COVID-19, even when respiratory symptoms are not severe, can trigger aHUS.

## Introduction

Atypical hemolytic uremic syndrome (aHUS) is a form of thrombotic microangiopathy (TMA) characterized by the triad of microangiopathic hemolytic anemia, thrombocytopenia, and acute kidney injury, and is caused by overactivation of the alternative complement pathway (1). Complement component 3 (C3) deficiency causes susceptibility to infections, whereas gain-of-function (GOF) variant in the *C3* gene causes susceptibility to aHUS (2). While aHUS can be triggered by various factors, there have been increasing reports of aHUS triggered by coronavirus disease 2019 (COVID-19) in recent years (3, 4). In this report, we present a 13-year-old boy who developed TMA following COVID-19 infection, and was eventually diagnosed with aHUS with a *C3* variant. This case highlights the potential of COVID-19 to trigger aHUS, particularly in individuals with underlying genetic predispositions.

## Case presentation

A 13-year-old Japanese boy with an unremarkable past medical history developed headache, malaise, and sore throat 2 days before presentation. The next day, he developed a cough, diarrhea, and gross hematuria. Upon visiting his local doctor, he was found to be positive for COVID-19 by rapid antigen test. He was noted to have thrombocytopenia with a platelet count of 19 × 10^9^/L. He was referred to a regional hospital the following day, where his laboratory results showed a further decline in platelet count (4 × 10^9^/L) and signs of microangiopathic hemolytic anemia. The creatinine level was 1.03 mg/dl, slightly elevated from his baseline creatinine levels (0.8–0.9 mg/dl). He was suspected of having TMA and was transferred to our hospital for further evaluation and management.

Upon admission to our hospital, his body temperature was 36.6°C, blood pressure was 120/69 mmHg, heart rate was 60 beats per minute, respiratory rate was 69 breaths per minute, and oxygen saturation was 96% on room air. He was fully conscious and well oriented. Physical examination was unremarkable, except for petechiae on the bilateral upper extremities. Notable results of initial laboratory analysis included unconjugated hyperbilirubinemia (T-Bil 5.7 mg/dl, D-Bil 0.2 mg/dl), elevated LDH (3,074 U/L), AST (137 U/L), BUN (32.6 mg/dl), creatinine (1.13 mg/dl), D-dimer (64.1 µg/ml), and CRP (2.41 mg/dl) levels, mild anemia with hemoglobin 10.5 g/dl, markedly decreased haptoglobin level (< 10 mg/dl), 4% schistocytes on the peripheral blood smear, severe thrombocytopenia (6 × 10^9^/L), and hematuria. ADAMTS13 activity was 85% of the normal control level. Complement analysis revealed normal CH50 and C4 levels, with a slightly decreased C3 level (70 mg/dl). A stool culture was negative for Shiga toxin-producing strains of *Escherichia coli* in the absence of prior antibiotic administration. Notably, the patient's father had a history of unexplained TMA following influenza infection that required plasma exchange for recovery when he was 36 years old, as well as an instance of gross hematuria following fever at the age of 16 years, the cause of which was not identified.

**Figure 1:**
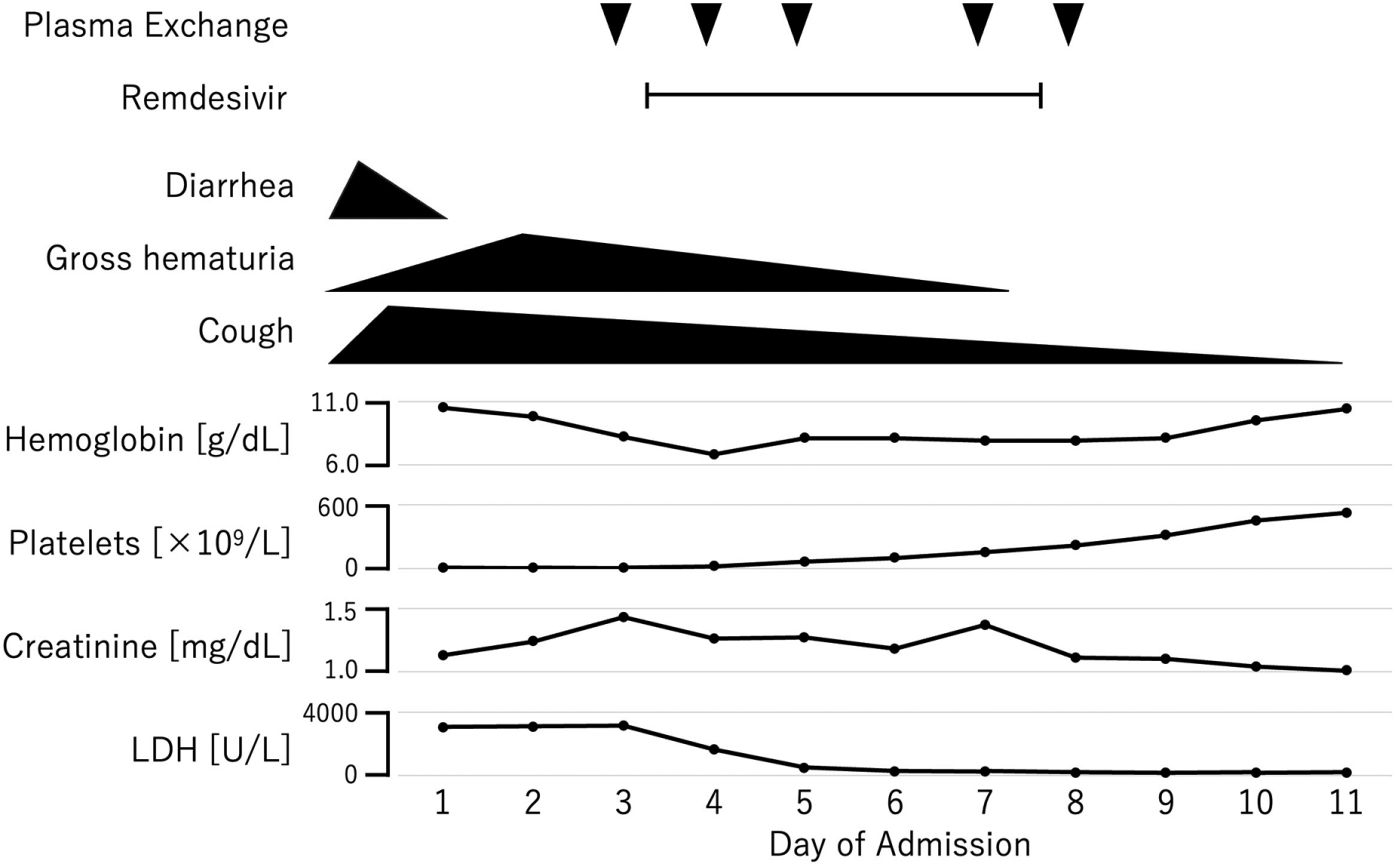
Clinical course of the patient.

By the third day of hospitalization, the anemia and thrombocytopenia had worsened, and the creatinine level rose to 1.43 mg/dl (Figure 1). At that point, the ADAMTS13 activity result had not yet been received, and thrombotic thrombocytopenic purpura (TTP) could not yet be excluded. Given the clinical presentation, both aHUS and TTP were still suspected. Consequently, plasma exchange therapy was initiated as a treatment for TMA, which could address both conditions. Plasma exchange was expected to be beneficial in the case of aHUS by removing abnormal complement system proteins and replenishing normal ones. In cases of anti-factor H antibody-associated aHUS, plasma exchange would also help remove anti-factor H antibodies. We excluded TTP after finding that the ADAMTS13 activity, which was obtained from a blood sample collected before the initiation of plasma exchange therapy, was not decreased. Because the platelet count and hemoglobin and creatinine levels were all improving, we did not start anti-C5 antibody therapy, and discontinued plasma exchange after five sessions. We intended to add anti-C5 antibody therapy if the patient's improvement was insufficient. Fortunately, the patient made a full recovery and was discharged on his 18th day in hospital. During the one year after discharge, he remained relapse free, despite having had influenza A once during that period.

The diagnosis of aHUS was confirmed by genetic analysis, which revealed a known pathogenic heterozygous *C3* GOF variant [NM_000064.4:c.3470T>C (p.Ile1157Thr)]. Anti-factor H autoantibody was negative in a plasma sample collected before the initiation of plasma exchange.

Genetic analysis of the patient's father revealed the same *C3* variant, suggesting that the unexplained TMA that the father had experienced was also aHUS.

## Discussion

Cases of TMA following COVID-19 have been increasingly reported (3, 4). In children, aHUS is the most commonly reported type of TMA, with 16 cases reported (Table 1). Only one patient presented with severe respiratory symptoms. Anti-factor H antibodies were identified in 5 cases. The majority of patients showed decreased C3 level and increased soluble C5b-9 level. Our patient also showed slightly decreased C3 level, but we could not measure the soluble C5b-9 level due to resource limitations. Most patients responded well to plasma exchange and/or anti-C5 therapy, but one patient required renal transplantation, and another patient required continued dialysis. TTP, though a rare cause of TMA in children, has also been observed in a small number of cases following COVID-19 infection (15, 16).

**Table 1:** Pediatric cases of aHUS following COVID-19.

Age, Sex	Presenting symptoms	Complement analysis	Treatment	Outcome
16 m, M (5)	Fever, DKA	C3 142 mg/dl, C4 19 mg/dl, sC5b-9 450 ng/ml	Eculizumab	Recovery
3y, F (6)	Fever, respiratory distress	C3 131 mg/dl, C4 10.9 mg/dl	Plasma exchange	Recovery
4y, M (7)	Anasarca, pallor, hypertension	C3 84 mg/dl, sC5b-9 4,220.3 ng/ml, anti-factor H antibodies(+)	Plasma exchange, prednisone, IV cyclophosphamide	Mild proteinuria
12y, M (7)	Abdominal pain, vomiting	C3 53 mg/dl, anti-factor H antibodies(+)	Plasma exchange, prednisone, mycophenol mofetil	Mild proteinuria
13y, M (7)	Abdominal pain, vomiting, hypertension	C3 48 mg/dl, sC5b-9 1,179.9 ng/ml, anti-factor H antibodies(+)	Plasma exchange, prednisone, mycophenol mofetil	Proteinuria
7y, M (7)	Abdominal pain, oliguria, hypertension	C3 69 mg/dl, sC5b-9 1,344.9 ng/ml, anti-factor H antibodies(+)	Plasma exchange, prednisone, mycophenol mofetil	CKD
10y, F (7)	Oliguria, pallor, hypertension	C3 64 mg/dl, sC5b-9 2,900.3 ng/ml, anti-factor H antibodies(+)	Plasma exchange, prednisone, IV cyclophosphamide	On dialysis
9y, M (8)	Fever, nausea, diarreha	C3, C4, sC5b-9 were all WNL	Supportive care	CKD
4 m, M (9)	Fever, mild respiratory symptoms	sC5b-9 456 ng/ml	Eculizumab	Proteinuria
4.5 m, M (9)	Fever, diarrhea, reduced drinking	sC5b-9 311 ng/ml	Eculizumab	Mild proteinuria
2y, F (10)	Diarrhea	C3 106 mg/dl	Supportive care	Recovery
21 m, M (11)	Fever, diarrhea, vomit	C3 87 mg/dl, C4 18 mg/dl, sC5b-9 345 ng/ml	Plasma exchange, ravulizumab	Recovery
2y, M (12)	Diarrhea, tonic-clonic seizures, oliguria, melanic stools	C3 71 mg/dl, C4 8 mg/dl	Plasma exchange, IVIG, eculizumab	Recovery
23 m, F (13)	Restlessness, vomiting, diarrhea	C3 49 mg/dl, C4 11 mg/dl	Plasma exchange, eculizumab	Recovery
9 m, M (13)	Vomiting, diarrhea, fever	C3 60 mg/dl, C4 5 mg/dl	Plasma exchange	Recovery
13y, F (14)	Fever, vomiting	NA	Eculizumab, plasma exchange	Renal transplantation

DKA, diabetic ketoacidosis; CKD, chronic kidney disease; WNL, within normal limits; IVIG, intravenous immunoglobulin; NA, not available.

Multisystem inflammatory syndrome in children (MIS-C) is a hyper-inflammatory disorder occurring 2–6 weeks after severe acute respiratory syndrome coronavirus 2 (SARS-CoV-2) infection. It consists of persisting fever, involvement of two or more organ systems, clinical severity requiring hospitalization, and laboratory evidence of inflammation. It has been reported that 20% of patients with MIS-C develop acute kidney injury (AKI) (17). MIS-C may also present with variable degree of hematuria (18). MIS-C should be included in the differential diagnosis when AKI or hematuria is present following COVID-19. Generalic ´ et al. reported a case of MIS-C that presented with hematuria and suggested that hematuria may be an early sign of MIS-C (19). Hematuria can be an early symptom in both MIS-C and TMA, making it an important sign that could prompt consideration of these conditions. When hematuria is observed following COVID-19, distinguishing between MIS-C and TMA becomes a critical diagnostic challenge. Findings suggestive of intravascular hemolysis (e.g., decreased haptoglobin) and schistocytes on the peripheral blood smear can help distinguish TMA, such as aHUS or TTP, from MIS-C. The distinction between these conditions is critical, as the management strategies differ significantly.

The *C3* GOF variant is a relatively rare cause of aHUS globally, but this variant, particularly the *C3* p.Ile1157Thr variant, is notably common in the Japanese population (20). This variant predisposes individuals to excessive complement activation by reducing the binding affinity of C3b to complement factor H (CFH) and/or membrane cofactor protein, or by conferring resistance to factor I-mediated inactivation of C3b (1). Patients with this genetic defect develop aHUS when complement activation becomes dysregulated, often in response to external triggers. While cases of aHUS with a *C3* variant have been reported following a variety of preceding events, recent reports have increasingly linked COVID-19 to the development of aHUS (Table 2).

**Table 2:** Triggering events of aHUS in patients with a *C3* variant.

#	Author, year	Age of onset	*C3* mutation	Preceding events
1	Lhotta, 2009 (21)	22y	R570Q	Pyelonephritis, cholecystitis, and a laparoscopic cholecystectomy
2	Al-Akash, 2011 (22)	1y	R570W	Influenza A
3	Brackman, 2011 (23)	4m-5m	R713W, G1094R	Respiratory tract infection, vaccination
4	Roumenina, 2012 (24)	9m-35y	R139W	Infection, oral contraception, postpartum, cocaine
5	Volokhina, 2012 (25)	18y-40y	K65Q	Kidney transplantation
6	Fan, 2013 (26)	8m-70y	R425C, I1157T	Upper respiratory tract infection, surgery
7	Bresin, 2013 (27)	2y-23y	R161W, H1464D	Infection, pregnancy
8	Matsumoto, 2013 (28)	1y-24y	I1157T	Influenza, common cold, infection, vaccine
9	Siomou, 2014 (29)	10m	I1157T	Febrile viral respiratory infection
10	Toyoda, 2016 (30)	9y	I1157T	Viral gastroenterocolitis, *Campylobacter jejuni*, bacterial pharyngitis
11	Hoeve, 2017 (31)	10y	R161W	Influenza B
12	Okano, 2018 (32)	23y	I1157T	Influenza A
13	Matsumoto, 2018 (33)	1y-21y	I1157T	Influenza, mumps, common cold, bacterial infection, vaccine
14	Han, 2019 (34)	4m	E1160K	Brief fever
15	Lumbreras, 2020 (35)	8y	S179P	Nonbloody diarrhea
16	Okabe, 2021 (36)	8y	I1157T	Fever and sore throat, followed by abdominal pain and diarrhea
17	Kim, 2021 (37)	23y	S562l	General malaise, abdominal pain, and diarrhea
18	Mat, 2021 (38)	39y	R161W	COVID-19
19	Sangeetha, 2021 (39)	2y	L180del, S1038G	Acute dysentery
20	Claes, 2023 (40)	38y	C481T	COVID-19 booster vaccination
21	Uwatoko, 2023 (41)	3y	I1157T	COVID-19
22	Jelicic, 2023 (42)	37y	K155Q	Illicit drug abuse

Our patient, who had a known pathogenic C3 GOF variant, developed aHUS following COVID-19, despite having had other viral infections without subsequent aHUS earlier in his life. This suggests that SARS-CoV-2 may have a unique capacity to activate the alternative complement pathway, particularly in individuals with underlying complement system abnormalities. Recent studies have shown that the SARS-CoV-2 spike protein activates the complement lectin pathway by interacting with mannose-binding lectin (MBL), a key component of the lectin pathway (43). It has also been shown that the SARS-CoV-2 N protein binds to mannose-binding protein-associated serine protease 2 (MASP-2) and activates the complement lectin pathway either directly or by potentiating MBL-dependent MASP-2 activation (44, 45). This results in the generation of C3 convertases, which can further activate the alternative complement pathway by cleaving C3 in plasma. In genetically predisposed individuals, this can lead to sustained and excessive alternative complement pathway amplification, resulting in the manifestation of aHUS.

Moreover, SARS-CoV-2 spike protein has been shown to compete with complement factor H (CFH) for binding to heparan sulfate on the cell surface, disrupting CFH's regulatory function (46). CFH normally limits complement activation by displacing Bb from C3 convertases and facilitating the cleavage of C3b by factor I. In the presence of the SARS-CoV-2 spike protein, this regulation is impaired, which can lead to excessive activation of the alternative pathway and the onset of aHUS. These mechanisms illustrate the potential for SARS-CoV-2 to trigger complement-mediated diseases, especially in individuals with inborn defect in complement regulation.

Inborn errors of immunity have been noted as a risk factor for severe COVID-19. For example, abnormalities in genes related to type I interferons (IFNs) have been reported to underlie the severe form of COVID-19 (47). Their phenocopies, autoantibodies to type I IFNs, have also been reported to be associated with cases of life-threatening COVID-19 pneumonia (48). Autosomal recessive deficiencies of *OAS1*, *OAS2*, and *RNASEL* have been reported as genetic etiological factors behind MIS-C, a rare and severe pediatric complication of COVID-19 (49). The present case report shows that patients with inborn errors of the complement system are also at higher risk for severe illness from COVID-19.

This case reinforces the emerging understanding that COVID-19, even in the absence of severe respiratory symptoms, can trigger aHUS, particularly in individuals with genetic predispositions such as the *C3* GOF variant. Clinicians should be vigilant for signs of aHUS in children with COVID-19, especially those with a family history of TMA or complement system abnormalities, as early diagnosis and intervention can lead to favorable outcomes.

## Data Availability

The original contributions presented in the study are included in the article/Supplementary Material, further inquiries can be directed to the corresponding author.
